# *Giardia lamblia* G6PD::6PGL Fused Protein Inhibitors Decrease Trophozoite Viability: A New Alternative against Giardiasis

**DOI:** 10.3390/ijms232214358

**Published:** 2022-11-18

**Authors:** Laura Morales-Luna, Beatriz Hernández-Ochoa, Víctor Martínez-Rosas, Gabriel Navarrete-Vázquez, Daniel Ortega-Cuellar, Yadira Rufino-González, Abigail González-Valdez, Roberto Arreguin-Espinosa, Adrián Marcelo Franco-Vásquez, Verónica Pérez de la Cruz, Sergio Enríquez-Flores, Carlos Martínez-Conde, Luis Miguel Canseco-Ávila, Fernando Gómez-Chávez, Saúl Gómez-Manzo

**Affiliations:** 1 Laboratorio de Bioquímica Genética, Instituto Nacional de Pediatría, Secretaría de Salud, Mexico City 04530, Mexico; 2 Posgrado en Ciencias Biológicas, Universidad Nacional Autónoma de México, Mexico City 04510, Mexico; 3 Laboratorio de Inmunoquímica, Hospital Infantil de México Federico Gómez, Secretaría de Salud, Mexico City 06720, Mexico; 4 Programa de Posgrado en Biomedicina y Biotecnología Molecular, Escuela Nacional de Ciencias Biológicas, Instituto Politécnico Nacional, Mexico City 11340, Mexico; 5 Facultad de Farmacia, Universidad Autónoma del Estado de Morelos, Av. Universidad 1001, Chamilpa, Cuernavaca 62209, Mexico; 6 Laboratorio de Nutrición Experimental, Instituto Nacional de Pediatría, Secretaría de Salud, Mexico City 04530, Mexico; 7 Laboratorio de Parasitología Experimental, Instituto Nacional de Pediatría, Secretaría de Salud, Mexico City 04530, Mexico; 8 Departamento de Biología Molecular y Biotecnología, Instituto de Investigaciones Biomédicas, Universidad Nacional Autónoma de México, Mexico City 04510, Mexico; 9 Departamento de Química de Biomacromoléculas, Instituto de Química, Universidad Nacional Autónoma de México, Mexico City 04510, Mexico; 10 Neurobiochemistry and Behavior Laboratory, National Institute of Neurology and Neurosurgery “Manuel Velasco Suárez”, Mexico City 14269, Mexico; 11 Laboratorio de Biomoléculas y Salud Infantil, Instituto Nacional de Pediatría, Secretaría de Salud, Mexico City 04530, Mexico; 12 Facultad de Ciencias Químicas, Campus IV, Universidad Autónoma de Chiapas, Tapachula City 30700, Mexico; 13 Laboratorio de Enfermedades Osteoarticulares e Inmunológicas, Sección de Estudios de Posgrado e Investigación, Escuela Nacional de Medicina y Homeopatía, Instituto Politécnico Nacional, Mexico City 07320, Mexico

**Keywords:** antiparasitic, inhibitors, molecular docking, giardiasis, antigiardial

## Abstract

Treatments to combat giardiasis have been reported to have several drawbacks, partly due to the drug resistance and toxicity of current antiparasitic agents. These constraints have prompted many researchers to investigate new drugs that act against protozoan parasites. Enzyme inhibition is an important means of regulating pathogen metabolism and has recently been identified as a significant alternative target in the search for new treatments. Glucose-6-phosphate dehydrogenase and 6-phosphogluconolactonase (G6PD::6PGL) is a bifunctional enzyme involved in the pentose phosphate pathway (PPP) in *Giardia lamblia* (*G. lamblia*). The *G. lamblia* enzyme is unusual since, unlike the human enzyme, it is a fused enzyme. Here, we show, through inhibition assays, that an in-house chemical library of 120 compounds and four target compounds, named CNZ-7, CNZ-8, CMC-1, and FLP-2, are potent inhibitors of the *G. lamblia* G6PD::6PGL fused enzyme. With a constant (*k*_2_) of 2.3, 3.2, and 2.8 M^−1^ s^−1^, respectively, they provoke alterations in the secondary and tertiary protein structure and global stability. As a novel approach, target compounds show antigiardial activity, with IC_50_ values of 8.7, 15.2, 15.3, and 24.1 µM in trophozoites from *G. lamblia*. Moreover, these compounds show selectivity against *G. lamblia*, since, through counter-screening in Caco-2 and HT29 human cells, they were found to have low toxicity. This finding positions these compounds as a potential and attractive starting point for new antigiardial drugs.

## 1. Introduction

Giardiasis is an intestinal disease caused by the parasite *Giardia lamblia* (*G. lamblia*), generally related to traveler’s disease [1]. The prevalence of giardiasis worldwide varies depending on its geographical location, from 2% to 3% in industrialized countries and 30% in low-income and developing countries [2]. Due to the high incidence of the disease throughout the world, the WHO has classified *G. lamblia* as the third most common biological agent causing diarrheal diseases, with more than 300 million clinical cases diagnosed annually [3]. Giardiasis can present itself in various clinical manifestations, within which asymptomatic patients are described, while it includes acute diarrhea or malabsorption syndrome in its most severe form [4,5]. The symptomatology in people with giardiasis is varied, since it depends on the individual factors of the patients, such as the immune status, the degree of virulence of the strain, as well as the dose of cysts ingested, and the degree of the duration of the parasitosis [6]. In chronic patients, poor intestinal absorption affects the assimilation of fats, vitamins A and B12, folic acid, and lactose [5,7,8]. The infection occurs mainly in children, with more common severe manifestations, contributing to malnutrition and growth retardation in patients [1].

**Figure 1:**
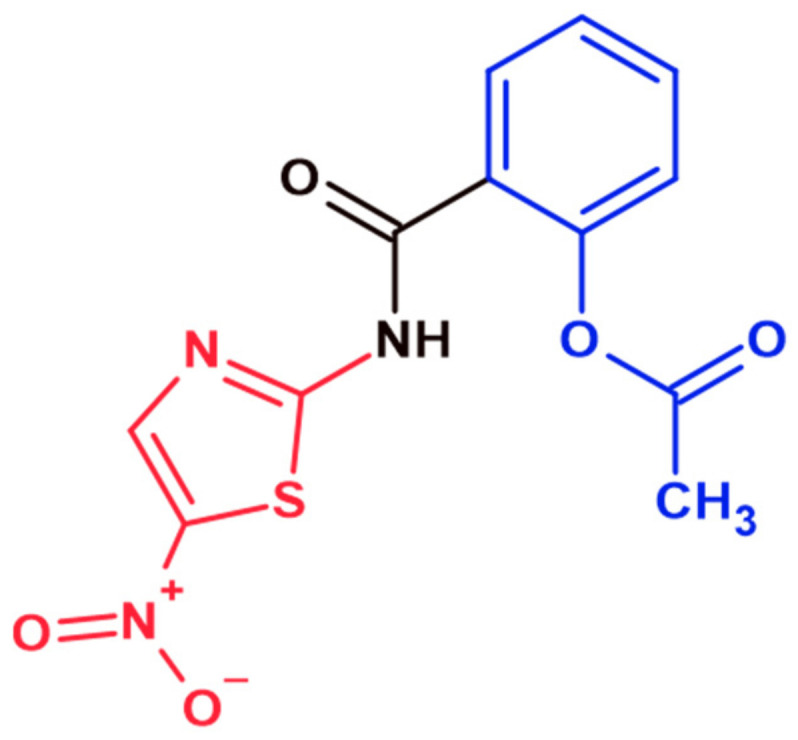
Nitazoxanide structure. This drug comprises two fragments, the 2-amino-5-nitrothiazole head (red color) and the acetylsalicylic tail (blue color), which are joined by an amide bridge. The chemical structure was generated with the ChemSketch program (Advanced Chemistry Development, Inc. Toronto, ON, Canada).

The conventional approach to treating giardiasis includes oral agents such as nitroimidazole derivates, albendazole, furazolidone, and nitazoxanide, which, in their active forms, cause generic alterations in proteins and changes in the helical structure of the parasite’s DNA, with strand breakage and loss of its functions [9,10,11]. However, the use of nitroimidazole derivates and other antigiardial drugs, exceedingly if prolonged, is associated with several side effects, including the disruption of normal intestinal flora, possible carcinogenicity, the so-called metronidazole-induced encephalopathy, which occurs during the period of drug administration and produces temporary neurological effects, and limited or no efficacy due to the emerging phenomenon of drug resistance. Thus, resistant strains of *G. lamblia* have been isolated from patients with refractory giardiasis and even from in vitro cultures [9]. Nitazoxanide is one of the most common drugs acting against major diarrhea-causing etiologic agents. It is a 5-nitrothiazole with broad-spectrum activity against protozoa, anaerobic bacteria, and viruses [12]. Nitazoxanide comprises two fragments, the 2-amino-5-nitrothiazole head and the acetylsalicylic tail, which are joined by an amide bridge (Figure 1). Therefore, the development of new antigiardial drugs is of concern because giardiasis is a persistent problem.

The pentose phosphate pathway (PPP) is one of the most critical pathways in *G. lamblia* metabolism because this pathway is the primary source of NADPH, which, by reducing oxidized glutathione, controls oxidative stress, participates in the reductive synthesis of fatty acids, and produces another molecule known as the ribose sugar, used for the synthesis of nucleic acids. In addition, other metabolites are also produced in PPP to feed the glycolytic pathway, such as glyceraldehyde-3-phosphate and fructose-6-phosphate, among others [13]. Moreover, the PPP is essential for the survival of various parasitic organisms, such as *Plasmodium falciparum* [14,15,16,17].

Several organisms have evolved gene organizations that allow the optimization of their metabolism through the combination of two or more genes, which results in fused enzymes with multiple functions [14,18]. Interestingly, glucose-6-phosphate dehydrogenase and 6-phosphogluconolactonase (G6PD::6PGL), enzymes of PPP in *G. lamblia*, have been described as fused enzymes [13,19]. They catalyze the first and second steps of the PPP, and this arrangement may be more efficient in parasites (*P. falciparum* and *G. lamblia*) than in the host, where these two enzymes’ activities occur in different molecules [14,20]. This remarkable structural difference between Giardia G6PD::6PGL and human G6PD makes it a potential target for the development of new targeted drugs.

This work aimed to test an in-house chemical library of 120 compounds to identify active molecules that can alter G6PD::6PGL activity, which could be proposed as new, effective therapeutic agents to treat giardiasis. Here, we investigate the effect of a set of bioactive compounds, including aromatic and non-aromatic heterocyclic compounds with heteroatoms such as nitrogen, oxygen, and sulfur, by inhibition assays with the recombinant GlG6PD::6PGL enzyme. We found four compounds (CNZ-7, CNZ-8, CMC-1, and FLP-2) that inhibited the activity of the enzyme; then, the second-order inactivation constants (*k*_2_) were determined. Furthermore, by spectroscopic methods, we determined that these compounds induced alterations in the protein’s secondary and tertiary structure. In addition, we demonstrated that the compounds displayed greater antigiardial activity and low toxicity on Caco-2 and HT29 cells concerning the commercial drug nitazoxanide. Finally, we also performed molecular docking to predict the potential binding sites of inhibitors on the GlG6PD::6PGL structure.

## 2. Results and Discussion

### 2.1. Purification of the Recombinant Protein G6PD::6PGL

The G6PD::6PGL fused enzyme from *G. lamblia* was purified, as previously reported by Morales-Luna et al. [19], using a Ni Sepharose high-performance affinity column. The purified protein was analyzed by 12% SDS-PAGE. As seen in Supplementary Figure S1, a single band with a relative molecular mass of 72 kDa was obtained, which agrees with the theoretical parameters according to the Expasy ProtParam tool (https://web.expasy.org/protparam/ (accessed on 24 June 2022) (Swiss Institute of Bioinformatics, Lausanne, Switzerland). Afterward, the 6xHis tag located in the N-terminal of the G6PD::6PGL protein was removed using the site-specific protease, the tobacco etch virus protease (TEVP), as previously reported [19,21]. The resulting protein was used immediately to perform all the assays involved in this study.

### 2.2. Selection of Inhibitors of G6PD::6PGL Fused Enzyme of Giardia lamblia

Low-throughput screening (LTS) assays have been widely used to identify enzyme inhibitors on the fused *Plasmodium falciparum* and *Trichomonas vaginalis* G6PD::6PGL proteins [22,23]. Based on the above, we decided to evaluate the effect in vitro of the compounds in the inactivation of the G6PD::6PGL enzyme. A library of 120 compounds with similar structures to the molecules previously reported by Preuss et al. [23] was tested at a final concentration of 400 μM. The results showed that four chemical compounds, named CNZ-7, CNZ-8, CMC-1, and FLP-2, reduced the activity of the fused G6PD::6PGL from 70% to 98% of the initial activity. CMC-1, a benzylidene-barbituric compound, showed a more significant percentage of inhibition, with 98%, followed by 5-nitrothiazoles FLP-2, CNZ-7, and CNZ-8, with 89%, 85%, and 75% of inhibition, respectively. Moreover, we examined the effect of these compounds on the activity of G6PD from *Homo sapiens* (HsG6PD) and observed that FLP-2 and CMC-1 reduced the initial activity by around 20% and 34%, while the CNZ-7 and CNZ-8 compounds reduced the activity of HsG6PD by 68% and 43%, respectively. It is important to note that although these four compounds inhibited both the fused G6PD::6PGL from *G. lamblia* and HsG6PD, the inhibition was higher in the parasite enzyme regarding the HsG6PD enzyme; this selectivity is marked mainly by the FLP-2 and CMC-1 compounds (Table 1). Moreover, it is essential to note that the selective inhibition of the FLP-2 and CMC-1 compounds over the fused G6PD::6PGL enzyme may help to improve the rational design of new drugs against this parasite without affecting the activity of HsG6PD.

### 2.3. In Vitro Screening of Fused G6PD::6PGL Inactivation

**Figure 2:**
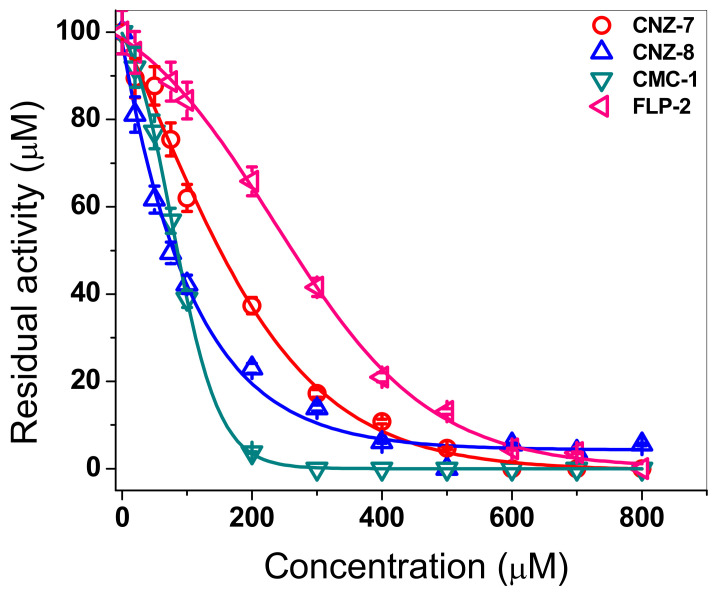
Effects of CNZ-7, CNZ-8, CMC-1, and FLP-2 chemical compounds on the activity of G6PD::6PGL enzyme. G6PD::6PGL protein was adjusted at 0.2 mg/mL and incubated in the presence of different concentrations of compounds (0–800 μM). After incubation, aliquots were withdrawn from the samples, and the residual activity was determined. The G6PD::6PGL without compounds was used to determine 100% activity. The assays were carried out in triplicate, and the data represent the mean ± standard error.

Since the four synthetic compounds showed more than 70% inactivation of the fused G6PD::6PGL enzyme from *G. lamblia* and inactivated at a lower proportion the human G6PD enzyme, we performed inactivation using different concentrations of these novel compounds to evaluate and analyze the concentration–activity effect. As seen in Figure 2, as the concentration of each of the compounds increased, a decrease in residual activity was observed after incubation for 2 h at 37 °C. The enzyme activity was abolished at 100% when the enzyme was incubated with 200 µM of CMC-1. In contrast, at the same concentration, the enzyme lost 80% of its initial activity in CNZ-8, and 60% and 40% in CNZ-7 and FLP-2, respectively. Moreover, from the plots of residual activity data, we calculated the IC_50_ values. The IC_50_ values determined were 70 µM, 80 µM, 150 µM, and 256 µM for CMC-1, CNZ-8, CNZ-7, and FLP-2, respectively (Figure 2). These results indicate that the enzyme lost its activity faster with the compounds CMC-1 and CNZ-8, showing a more significant negative effect on the catalytic activity of G6PD::6PGL at low concentrations. Finally, it is important to mention that, previously, Ramirez-Nava et al. [24] reported 274 µM as the IC_50_ value for the CNZ-7 compound on HsG6PD, while the IC_50_ value for the fused enzyme G6PD::6PGL was 150 µM, indicating that this compound has a stronger inhibitory property towards the *G. lamblia* enzyme regarding the human G6PD enzyme.

An inactivation assay with the above conditions was performed with the drug nitazoxanide and the compound barbituric acid (Table 1); we found that barbituric acid does not have the property of inhibiting the GlG6PD::6PGL enzyme (IC_50_ > 500 µM), while nitazoxanide shows an IC_50_ of 78 µM. This result could indicate that the GlG6PD::6PGL enzyme is another therapeutic target for this drug, which contributes to its antiparasitic action; however, this should be studied in the future.

**Figure 3:**
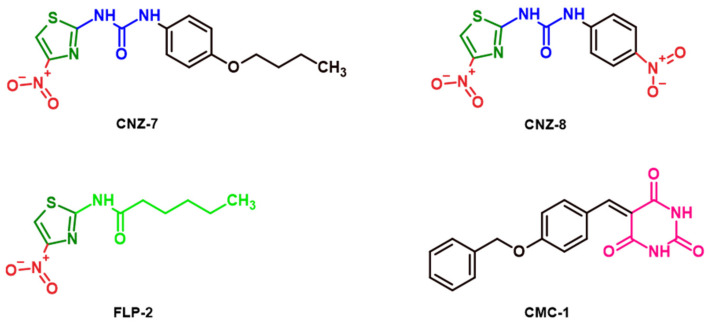
The chemical structures of the compounds that displayed G6PD enzyme inhibition activity >70%. CNZ-7, CNZ-8, and FLP-2 are nitazoxanide derivatives. The thiazole ring is shown in green the nitro group is shown in red; the urea group is shown in blue; the hexanamide substituent is shown in light green; the barbituric ring is shown in pink. The chemical structures were generated with the ChemSketch program (Advanced Chemistry Development, Inc. Toronto, ON, Canada).

The differences in the IC_50_ values between these chemical compounds were probably due to the structural differences that each compound presents, as was observed in the low-throughput screening (LTS) assay. Interestingly, the four compounds tested here inhibited the activity of HsG6PD to different extents, and the inhibitory effect revealed a concentration-dependent inhibition (Figure 3). The CNZ-7, CNZ-8, and FLP-2 compounds are derivatives of nitazoxanide drugs. Their structure has a nitrothiazole ring, CNZ-7 and CNZ-8 have a urea group, while FLP-2 has a hexanamide substituent. CMC-1 is a benzylidene barbiturate compound.

### 2.4. Rate Inactivation Constants (k_2_) of Selected Compounds

**Figure 4:**
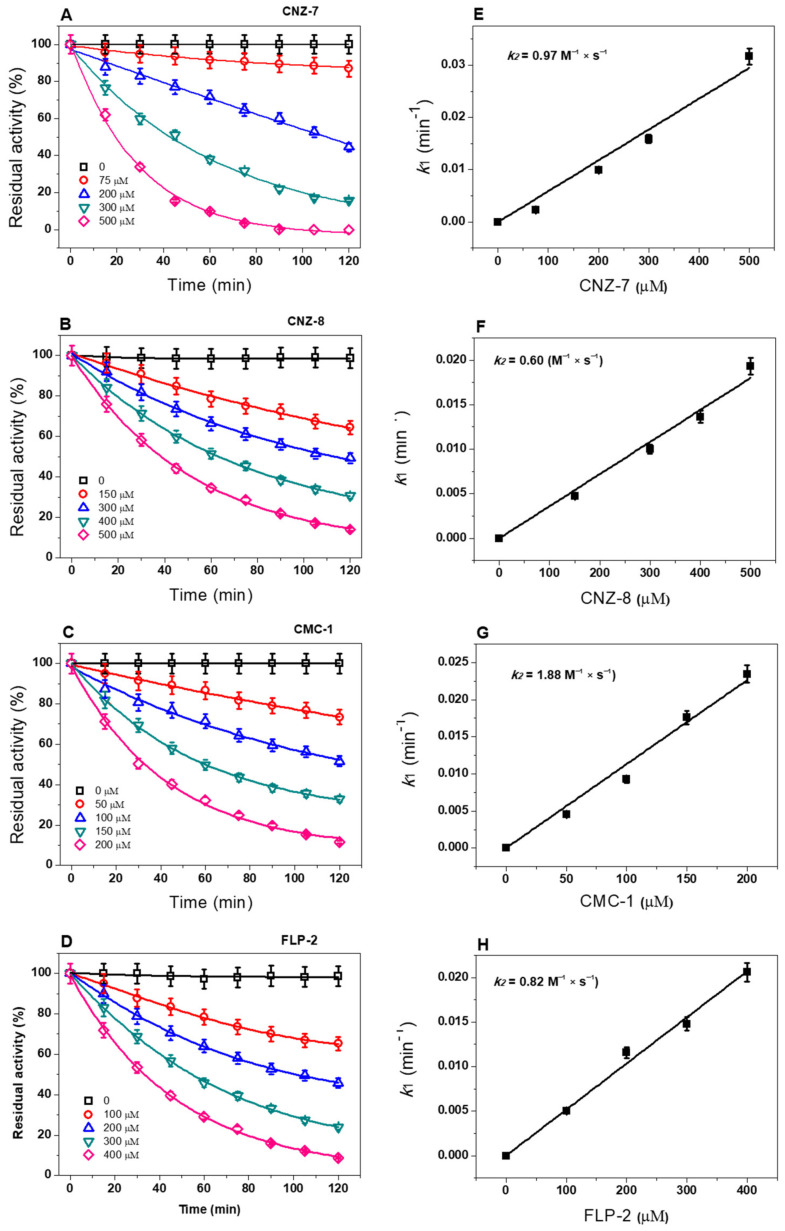
Inactivation of GlG6PD::6PGL by CNZ-7, CNZ-8, CMC-1, and FLP-2 chemical compounds. G6PD::6PGL enzyme was adjusted to 0.2 mg/mL and was incubated with different concentrations of (**A**) CNZ-7, (**C**) CNZ-8, (**E**) CMC-1, and (**G**) FLP-2 at 37 °C. At the indicated times, aliquots were withdrawn from the samples and assayed for residual activity. Initial velocity was fitted to determine each compound’s pseudo-first-order inactivation constants (*k_1_*). The second-order rate constant values of inactivation (*k*_2_) of each of the compounds (**B**) CNZ-7, (**D**) CNZ-8, (**F**) CMC-1, and (**H**) FLP-2 were obtained by fitting the calculated *k_1_* value versus the concentration of the corresponding compound and adjusting to a linear regression model. All experiments were performed in triplicate. The values represent the mean ± standard deviation from three independent experiments, with standard errors lower than 5%.

Based on the inactivation data, we determined the second-order inactivation constants (*k*_2_) of the four compounds, representing the rate of formation of the enzyme–inactivator complex. First, we determined the pseudo-first-order inactivation constants (*k*_1_) by measuring the initial velocities at five fixed compound concentrations between 0 and 120 min intervals. As seen in Figure 4, the four synthetic compounds showed a single-exponential decay in time-course inactivation and had a negative effect on the catalytic activity. The calculated *k_2_* values for CNZ-7, CNZ-8, CMC-1, and FLP-2 were 0.97, 0.60, 1.88, and 0.82 M^−1^ s^−1^, respectively. It is important to mention that CMC-1 formed the enzyme–inhibitor complex faster (*k_2_* value of 1.88 M^−1^ s^−1^), followed by the CNZ-7 and FLP-2 compounds. These results indicate that CMC-1 has the best *k_2_* value and can bind faster with the G6PD::6PGL fused enzyme to form the enzyme–inhibitor complex and thus inactivate the enzyme. The four compounds selected in this study are inhibitors for the fused G6PD::6PGL protein and could be used as new candidate drugs against *G. lamblia*. Furthermore, the present work describes the first approach to the identification of compounds that inhibit the G6PD::6PGL enzyme, thus laying the groundwork for the study of G6PD::6PGL as a potential target for rational drug design.

### 2.5. Circular Dichroism (CD) Assay

**Figure 5:**
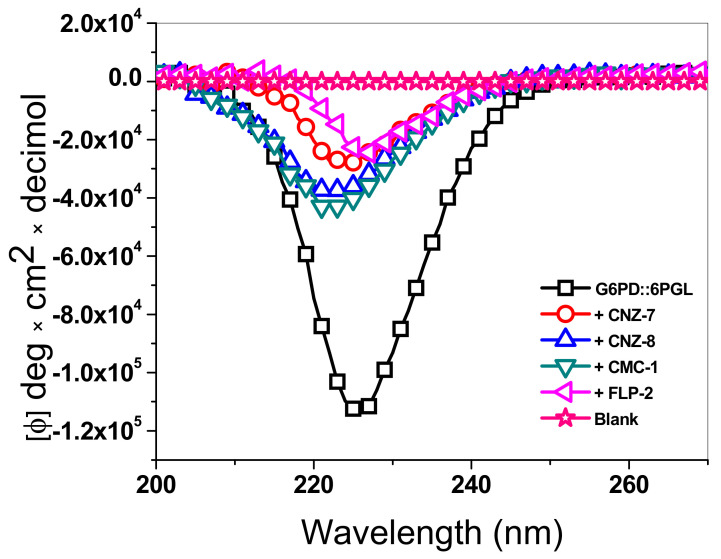
Far-UV circular dichroism (CD) spectra of GlG6PD::6PGL. The selected compounds at the IC_50_ concentration of CNZ-7, CNZ-8, CMC-1, and FLP-2 were incubated with 0.4 mg/mL of GlG6PD::6PGL protein for 2 h at 37 °C before measurement. The spectra in the far-UV region from 200 to 260 nm were obtained. The experiment is representative of triplicate experiments.

Due to the G6PD::6PGL enzyme from *G. lamblia* showing a loss of catalytic activity in the presence of the selected compounds, circular dichroism (CD) assays were performed in the absence or presence of the IC_50_ concentration of each compound to determine if the loss of catalytic activity was due to alterations in the secondary structure caused by the interaction of the compounds with the protein. As seen in Figure 5, the spectrum of the G6PD::6PGL protein free of the compound showed a defined negative absorption peak at 222 nm, corresponding to α-helices. In contrast, in the presence of the four selected compounds, alterations in the intensity pattern were observed concerning the protein in the absence of the compounds. Thus, the four chemical compounds altered the secondary structure of G6PD::6PGL because the spectra were closer to the blank. The compounds that most altered the secondary structure were FLP-2 and CNZ-7, with a loss of around 80% with respect to the GlG6PD::G6PD protein free compound; CNZ-8 and CMC-1 had a loss of 70% on the secondary structure of the protein that was observed with respect to the protein free of compounds. These results indicate that the loss of catalytic activity on the G6PD::6PGL enzyme of *G. lamblia* is due to alterations in the secondary structure caused by the interaction of the compounds with the protein.

### 2.6. Intrinsic and Extrinsic Fluorescence Assays

**Figure 6:**
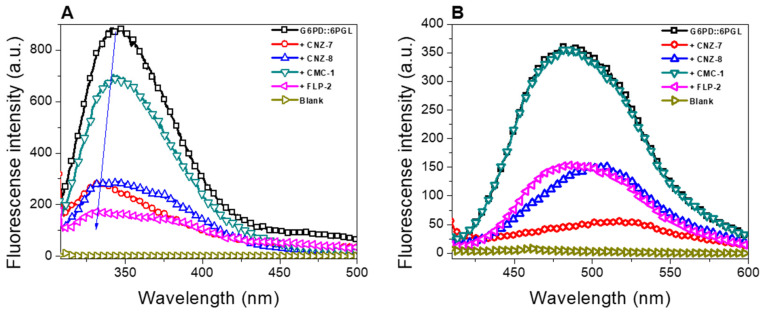
Fluorescence emission spectra of G6PD::6PGL enzyme. (**A**) Intrinsic fluorescence spectra of the G6PD::6PGL enzyme, and (**B**) extrinsic fluorescence spectra ANS assays, free or with CNZ-7, CNZ-8, CMC-1, and FLP-2 chemical compounds. The selected compounds at the IC_50_ concentration were incubated with 0.1 mg/mL of G6PD::6PGL protein for 2 h at 37 °C before measurement. The data are the mean of at least four independent experiments.

Another approach to evaluating the effects of the four selected compounds on the G6PD::6PGL protein was monitoring the changes in tertiary structure via the intrinsic fluorescence properties of the protein. Based on the above, we evaluated the intrinsic fluorescence of the eight tryptophan residues contained in the G6PD::6PGL/monomer in the absence or presence of the IC_50_ concentration of each compound. The intrinsic fluorescence of the native protein free of compounds showed a peak with a spectral center at 343 nm, with a maximum intensity of 874 arbitrary units (a.u.) (Figure 6A), while, in the presence of the compounds, there was a reduction in the maximum peak of fluorescence of 20–80% of the initial signal intensity (Figure 6A). As seen in Figure 6A, the four selected compounds lowered the intrinsic fluorescence intensity on the parasite G6PD::6PGL protein from *G. lamblia*. The compound that induced the most significant changes in the tertiary structure of the protein was FLP-2, with a maximum intensity of fluorescence of 171 a.u., which means a decrease to 80% regarding the enzyme free of the compound. CNZ-7 and CNZ-8 induced the second most significant alteration in the native enzyme, with a loss of intrinsic fluorescence of 67% regarding the compound-free native enzyme, where the maximum fluorescence intensity was 276 a.u. Finally, CMC-1 showed a maximal fluorescence intensity of 695 a.u, indicating a reduction of 20%, compared to the G6PD::6PGL enzyme free of the compound. Moreover, a blue shift in the fluorescence intensity was observed in the presence of the CNZ-7, CNZ-8, and FLP-2 compounds, indicating that the protein aggregates and buried the tryptophan residues. The results obtained in this work indicated that the four compounds showed alterations in the global stability of the G6PD::6PGL protein and that the binding of the compounds caused a change in the tryptophan residues’ microenvironment, altering the secondary and three-dimensional (3D) structure, provoking an inhibitory effect on the catalytic activity.

Finally, we performed structural assays that allowed us to evaluate alterations on the tertiary structures provoked by the binding of the compound to the enzyme; we used the fluorescence of the ANS assay, which has been widely used to determine alterations of proteins in the presence of an external agent [22,24]. The enzyme was incubated with the four compounds and the extrinsic fluorescence was measured. As seen in Figure 6B, the fused G6PD::6PGL enzyme in the presence of the CNZ-7, CNZ-8, and FLP-2 compounds showed a decrease in fluorescence intensity compared to the protein in the absence of compounds; meanwhile, the CMC-1 compound showed the same pattern in fluorescence intensity concerning the native enzyme without compounds. The CNZ-7 compound was the one that most affected the 3D structure of the protein, with a decrease of 85% in fluorescence intensity (52 a.u.), followed by the CNZ-8 and FLP-2 compounds (155 a.u.), where a decrease in fluorescence intensity of 56% was observed regarding the native G6PD::6PGL enzyme from *G. lamblia* free of compounds. These decreases in fluorescence intensity of more than 85% agree with the previously observed intrinsic fluorescence intensity, where a blue shift was observed, indicating that the enzyme aggregates and does not expose buried hydrophobic regions after treatment with the selected compounds. Consequently, the ANS probe can no longer bind.

Regarding these results, it is interesting to note that, in both functional and structural analysis, the four selected compounds caused a loss of catalytic activity and alterations in the secondary and 3D structure of the fused G6PD::6PGL protein, making them good candidates to be applied as experimental antigiardial drugs

### 2.7. Molecular Docking Method to Estimate the Binding Energy

Based on the biochemical assays, the most active compounds (CNZ-7, CNZ-8, CMC-1, and FLP-2) were selected for further computational studies to explore their putative mechanism of action at the molecular level. Therefore, a molecular docking study was performed to predict the molecular interactions between a target protein and the compounds.

**Figure 7:**
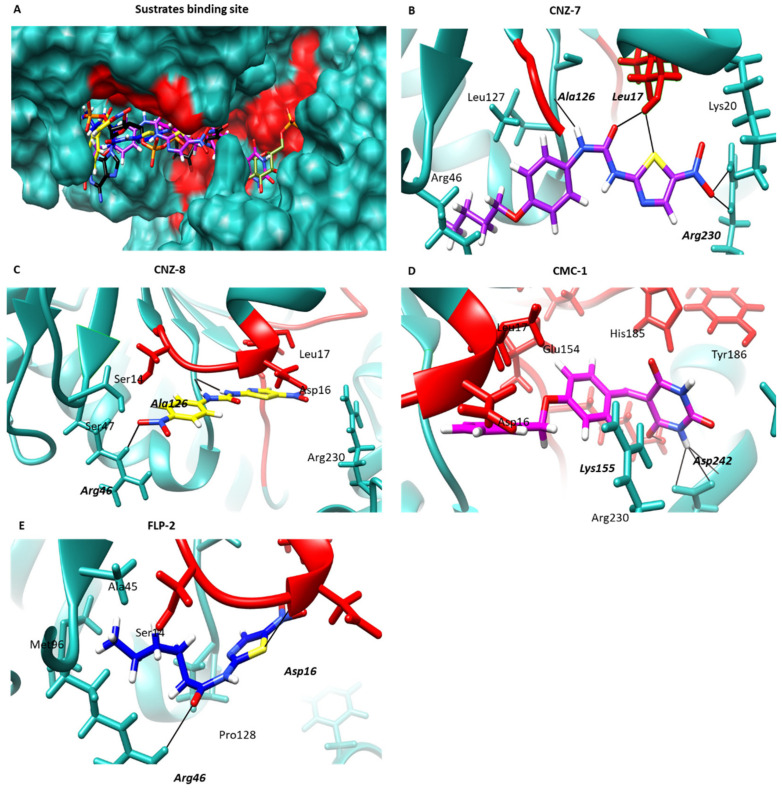
Molecular docking of the compounds on the G6PD::6PGL model. (**A**) General view of the predicted binding poses of CNZ-7 (purple), CNZ-8 (yellow), CMC-1 (pink), and FLP-2 (blue) in the binding pocket of substrates. The NADP^+^ and G6P molecules are shown in black and olive-green colors. (**B**) Closer view of the binding interactions of CNZ-7, (**C**) CNZ-8, (**D**) CMC-1, and (**E**) FLP-2. H-bonds are represented as black lines, and the amino acid residues are shown in bold and italic letters.

Molecular docking suggested that the four compounds have the potential to internalize in the vicinity of the substrate-binding site of the G6PD domain. The predicted binding poses of CMC-1 are characterized by being close to both the NADP^+^ and G6P substrates, while CNZ-7, CNZ-8, and FLP-2 were only found near the NADP^+^ interaction zone (Figure 7A). In the binding models, CNZ-7 and CMC-1 are the compounds that showed the most favorable free energy of binding, with scores of −7.47 and −7.11 kcal/mol, respectively, in this zone (Table 2). CNZ-7 forms two H-bond interactions between the nitro group of the compound and Arg230, a H-bond with the urea group and Ala126, and two H-bonds between the urea group and Leu17 (Figure 7B); the latter amino acid is part of the nucleotide-binding fingerprint (GxxGDLA). In addition, CNZ-8 forms three H-bond interactions, where the nitro and urea groups interact with Arg46 and Ala126, respectively (Figure 7C), showing a score of −7.06 kcal/mol (Table 2). Regarding the compound CMC-1, the most stable predicted binding position forms three H-bonds between the barbituric ring and Asp242 and one H-bond with Lys155 (Figure 7D). It is important to mention that the Lys155 is part of the conserved sequence, EKPxG (residues 154–158), which is critical for the correct positioning of the substrate (G6P) and coenzyme (NADP^+^) during the enzymatic reaction [25]. Finally, FLP-2 forms two H-bonds with Asp16 and Arg46 and with free energy binding of −6.55 kcal/mol; this compound had the lowest score value and, therefore, was the least stable in this zone. Docking results suggest that 5-nitrothiazole compounds CNZ-7, CNZ-8, and FLP-2 alter the correct positioning of the NADP^+^ substrate, while benzylidene-barbituric compound CMC-1 alters the environment of the binding site of both the NADP^+^ and G6P substrates; this probably causes CMC-1 to affect the activity of the enzyme G6PD::6PGL to a greater degree, which is related to the value of *k_2_* determined for this compound (1.88 M^−1^ s^−1^), which was the highest constant, followed by CNZ-7 (0.97 M^−1^ s^−1^).

**Figure 8:**
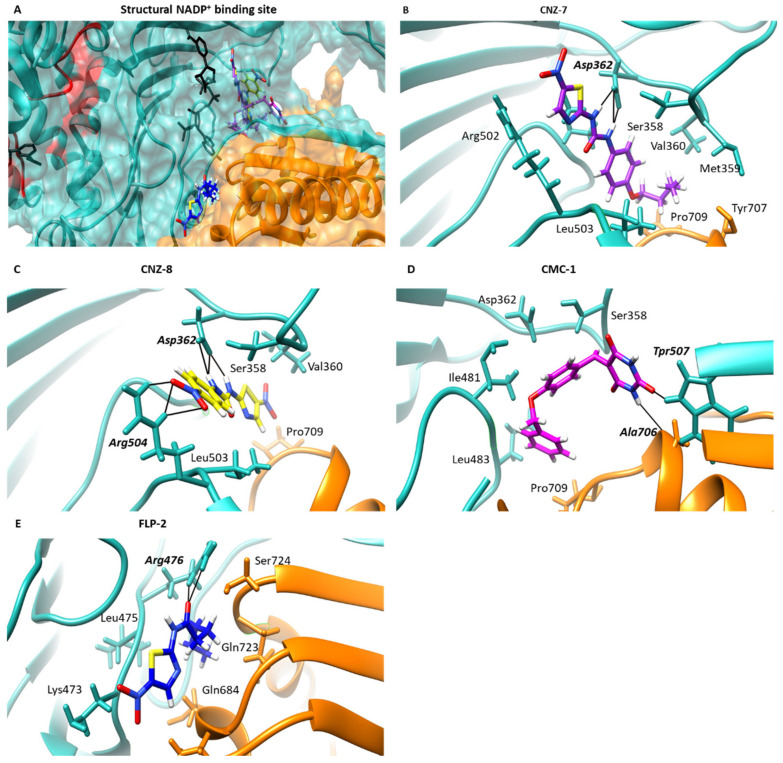
Molecular docking of the compounds on the G6PD::6PGL model. (**A**) General view of the predicted binding poses of CNZ-7 (purple), CNZ-8 (blue), CMC-1 (pink), and FLP-2 (blue) in the binding pocket of the structural NADP^+^ binding site. The NADP^+^ is shown in red color. (**B**) Closer view of the binding interactions of CNZ-7, (**C**) CNZ-8, (**D**) CMC-1, and (**E**) FLP-2. H-bonds are represented as black lines, and the amino acid residues are shown in bold and italic letters.

Another zone of interaction for the four compounds was found near the structural NADP^+^ binding site, whose zone is close to the 6PGL domain (Figure 8A). The main protein–ligand contacts of compounds were with Ser358, Asp362, Ile481, Arg504, and Pro709. CNZ-7 showed the most favorable free energy of binding, with a score of −8.62 kcal/mol, and formed three H-bonds with Arg362 (Figure 8B), while CNZ-8 is the compound that formed the most H-bonds, where six interactions were predicted; this included three H-bonds with Asp362 and three with Arg504 (Figure 8C). CMC-1 formed one H-bond with Ala706 (Figure 8D), and FLP-2 formed two H-bonds with Arg476 (Figure 8E). This interaction zone is close to the 6PGL domain of the protein, so, if the compounds bind near this zone, they probably cause the global stability of the protein to be altered, which was confirmed by the results found for DC, FI, and FE. Moreover, in a previous work, we predicted the possible structural NADP^+^ binding site in G6PD::6PGL, which is located between the Rossmann fold of G6PD and 6PGL, and it was observed through experimental trials that the G6PD::6PGL protein is stabilized by the NADP^+^ molecule [21]. Hence, the presence of the compounds probably negatively affects the stability of the enzyme and consequently decreases the activity, which agrees with our experimental results.

### 2.8. Concentration-Dependent Effect of Compounds on Giardia lamblia Trophozoites

**Figure 9:**
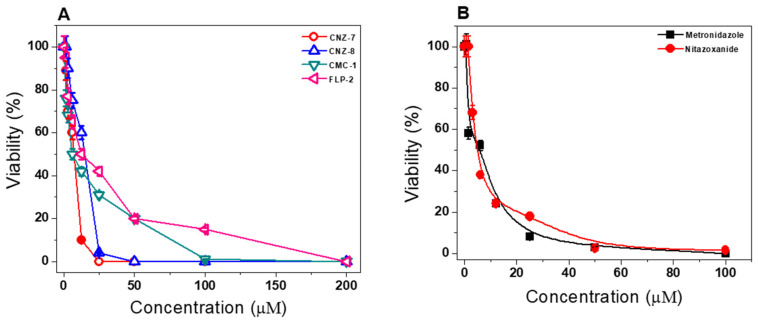
Antigiardial activity of the (**A**) CNZ-7, CNZ-8, CMC-1, and FLP-2 compounds, and (**B**) metronidazole and nitazoxanide on *G. lamblia*. The trophozoites were incubated in the presence of compounds for 48 h at 37 °C with increasing concentrations of each of the compounds, and later the viability of the trophozoites was determined. The values represent mean ± standard deviation from three independent experiments, and standard errors of the mean were lower than 5%.

To determine the antigiardial activity of the CNZ-7, CNZ-8, CMC-1, and FLP-2 compounds, we realized concentration-dependent curves to assess the viability of trophozoites from *G. lamblia* after incubation with different concentrations of each of the compounds. Figure 9A shows the curves of trophozoites’ viability obtained after exposure, and the IC_50_ values were calculated. The 5-nitrothiazole derivatives (CNZ-7, CNZ-8, and FLP-2) showed the best antigiardial activity, with IC_50_ values of 8.7, 15.2, and 15.3 µM, respectively, while the benzylidene-barbituric derivative CMC-1 showed an IC_50_ value of 24.1 µM. In addition, the thiazole ring is a heterocyclic nucleus widely exploited in pharmaceutical chemistry to design new drugs implicated in a wide variety of pathophysiological conditions [26,27]. The giardicidal activity shown by compounds CNZ-7, CNZ-8, and FLP-2 is probably also due to the presence of the nitro group in its structure, which could be involved in the redox metabolism of the parasite, affecting the survival of the trophozoites. In addition, this effect could be explained by the importance of the G6PD enzyme for cell metabolism, since it contributes to the redox balance and to the nucleotide and lipid biosynthesis pathways, which is in accordance with studies where authors have highlighted the importance of the enzyme in apoptosis and other processes in cancer, evaluating the use of inhibitory molecules in the enzyme and resulting in the death of cancer cells [28]. Following this line of research, different study groups have proposed the enzyme as a potential target in treating various ailments, among which are those caused by parasites [29]. The analysis of these pharmacological targets indicates the importance of this protein in all areas of disease, but also the growth of new first-class mechanisms, including parasitic diseases [16].

Regarding the mechanism proposed for nitazoxanide, the most widely accepted mechanism of nitazoxanide is believed to be the disruption of the energy metabolism by inhibiting the pyruvate:ferredoxin/flavodoxin oxidoreductase (PFOR) cycle [30,31]. Alternative mechanisms of action are the inhibition of protein disulfide isomerase [32] and interaction with *Giardia lamblia* nitroreductases (GlNR1, GlNR2) [33,34].

Metronidazole and nitazoxanide, which are drugs currently used to treat giardiasis, were used as a positive control. As shown in Figure 9B, metronidazole and nitazoxanide showed a lower IC_50_ value than the 5-nitrothiazole derivatives (CNZ-7, CNZ-8, and FLP-2).

It is important to note that the CNZ-7, CNZ-8, and FLP-2 compounds have a nitro group in their structure. Although the nitro group is considered to be a versatile and unique functional group in medicinal chemistry, it has also been well evidenced that drugs containing nitro groups can induce severe toxicity [35]. However, organ-selective toxicity with nitroaromatic compounds is also the basis of chemotherapy and results in the poisoning of bacteria, parasites, or tumor cells without harming the host organism or normal cells [36]. Therefore, the following assay tested the cytotoxicity of the four compounds using, as a model of the intestinal epithelium, a culture of cell lines Caco-2 and HT29.

### 2.9. Cytotoxic Effect of Compounds on Human Intestinal Caco-2 and HT29 Cells

**Figure 10:**
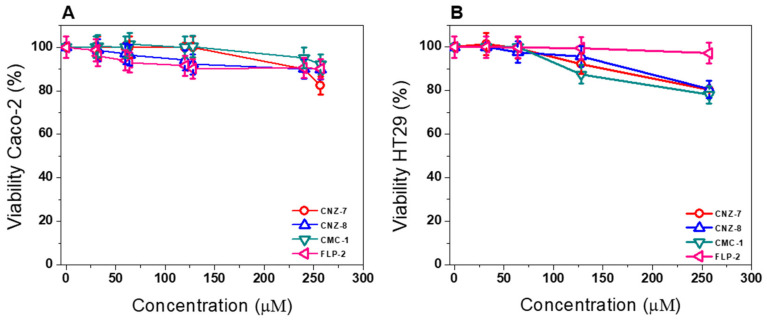
Cytotoxic effect of compounds on Caco-2 and HT29 cells via concentration–response curves for cell viability of (**A**) Caco-2 cells and (**B**) HT29 cells treated with CNZ-7, CNZ-8, FLP-2, and CMC-1. An XTT cell proliferation assay determined the viability. The values represent the mean ± standard deviation from three independent experiments, with standard errors lower than 5%.

Subsequently, we performed an assay to determine the cytotoxic effect of CNZ-7, CNZ-8, CMC-1, and FLP2 on two human intestinal cell lines as a model of the intestinal epithelium. We determined the concentration-dependent effects of the compounds on Caco-2 and HT29 cells viability, and the CC_50_ values (the concentration of the compounds that killed 50% of the cells) were calculated (Table 3). The compounds had low toxicity toward Caco-2 and HT29 eukaryotic cells, even at the highest concentrations tested (250 µM); the Caco-2 cell viability decreased by approximately 20% with 250 µM of CNZ-7 and by 25% with HT29 (Figure 10). Several studies have focused on the inhibition of human G6PD as a pharmacological target for the treatment of a variety of pathological conditions, including cancer and parasitic infections [37]. These results are significant because one of the main problems in developing new drugs is the high toxicity of the compounds towards mammalian cells, in such a way that the compounds studied in this work could be proposed as new antigiardial drugs for further study.

In the literature, the cytotoxicity of metronidazole and nitazoxanide has been reported in the HT29 and Caco-2 cell lines, with IC_50_ values of 265.9 µM [38] and 19 µM, respectively, for metronidazole [39]. Meanwhile, for nitazoxanide, results included an IC_50_ > 50 µM for HT29 [40] and 26.8 µM for Caco-2 [41]. Although CNZ-7, CNZ-8, CMC-1, and FLP-2 were less potent than nitazoxanide in killing the *G. lamblia* parasite, their cytotoxicity was much lower as well; the Caco-2 cell viability decreased by approximately 20% with 250 µM of CNZ-7, CNZ-8, and CMC-1, and 25% on HT29. Based on the selectivity index (SI), the data shown in Table 3 indicate that the compounds exhibit a higher degree of cytotoxic selectivity in *G. lamblia* than nitazoxanide.

In order to estimate the pharmacokinetic profiles of compounds CNZ-7, CNZ-8, FLP-2, CMC-1, NTZ, and barbituric acid, we used the predictor ADMETLab 2.0 (https://admetmesh.scbdd.com/ (accessed on 28 October 2022)) [42] to calculate the in silico ADMET profile (Table 4).

In the calculations of the pharmacokinetic profile, the compounds CNZ-7, CNZ-8, FLP-2, and CMC-1 presented high values of intestinal absorption, and all compounds, including NTZ and barbituric acid, have a medium to high probability of crossing the BBB. The distribution parameters calculated included the binding to plasmatic proteins, whose optimal value is <95%, and the compound CMC-1 was the only one that did not meet this parameter. Metabolic stability was calculated, and all compounds except for compound CNZ-7 had low values of substrates of the main metabolizing enzymes in the body, particularly CYP3A4 and CY2D6. Continuing with the ADMET calculations, excretion parameters were estimated; some of the compounds presented satisfactory clearance values, except for the compounds CNZ-8 and CMC-1, which presented low values, as well as NTZ and barbituric acid; all the compounds presented a long half-life (>3 h). Regarding the toxicity calculations, all the compounds presented a low probability of hERG channel blockade, indicating that they are non-cardiotoxic molecules [43]. For the calculation of acute oral toxicity in rats, all the compounds showed low toxicity, except the nitrothiazole FLP-2, which, similarly to its congener NTZ, showed some degree of toxicity. It is worth mentioning that all the nitrothiazole compounds, even NTZ, presented a high probability of being carcinogenic, and this effect is contributed by the nitro group in the compounds. It is well known that drugs containing nitro groups can induce severe idiosyncratic toxicity, and this is definitely the reason, in many cases, for their being avoided in drug design, considered as a structural risk. However, the nitro group is also considered as both a pharmacophore and a selective toxicophore associated with organ-selective toxicity [44], whereas barbituric acid and its surrogate CMC-1 lack these types of toxicity.

## 3. Materials and Methods

### 3.1. Overexpression and Purification of the Fused G6PD::6PGL Protein from Giardia lamblia

The study was carried out with the recombinant protein G6PD::6PGL from the *Giardia lamblia* WB strain (ATCC number 30957) and was overexpressed in the *E. coli* strain BL21(DE3)Δ*zwf*::kan^r^, which contains the plasmid with the *g6pd*::*6pgl* gene insert. Overexpression was induced with 0.3 mM isopropyl-β-D-1-thiogalactopyranoside (IPTG) (Thermo Fisher Scientific, Hudson, NH, USA), following the procedure reported by Morales et al. [19]. The protein was purified by the immobilized nickel metal affinity chromatography method and using Profinity^TM^ IMAC resin (Biorad, Hercules, CA, USA), following the method reported by Morales et al. [21]. Briefly, the resin was previously equilibrated with equilibrium buffer (50 mM Tris, 50 mM NaCl, pH 8). The protein was loaded onto the resin at a flow rate of 15 mL/h and washed with 5 column volumes of equilibrium buffer plus 25 mM imidazole. Later, the G6PD::6PGL protein was eluted using a 250 mM imidazole gradient. The presence of the active protein in the eluted fractions was determined by measuring the activity of the G6PD domain, following the spectrophotometric reduction at 340 nm of the NADP^+^ using a standard reaction mixture (100 mM Tris–HCl buffer, 30 mM MgCl_2_, 250 µM glucose-6-phosphate, and 250 µM NADP^+^, pH 8.0). Protein purity was verified by sodium dodecyl sulfate–polyacrylamide gel electrophoresis (SDS-Page) in denaturing conditions using the Kaleidoscope molecular mass marker (Biorad, Hercules, CA, USA). Protein concentration was determined by Lowry’s assay [45], using bovine serum albumin as a standard.

### 3.2. Functional Studies of the G6PD::6PGL Fused Enzyme Exposed to the Compounds

#### 3.2.1. Screening of In-House Chemical Library Compounds on the Activity of the Enzyme G6PD::6PGL of *G. lamblia*

The effects of 120 organic molecules from the in-house chemical library of compounds, which were synthesized in the Medicinal Chemistry Laboratory at the Pharmacy Faculty of the Autonomous University Morelos State, include mainly aromatic and non-aromatic heterocyclic compounds with heteroatoms such as nitrogen, oxygen, and sulfur, which were evaluated as possible specific inhibitors of G6PD::6PGL. The assay was carried out following what was reported by Martinez-Rosas et al. [22]; each compound was dissolved in dimethyl sulfoxide (DMSO), and the recombinant enzyme G6PD::6PGL (0.2 mg/mL) was incubated with the compounds at a concentration of 400 µM, for 2 h, at 37 °C. The residual enzymatic activity of the G6PD domain was measured following the spectrophotometric NADP^+^ reduction at 340 nm with the standard reaction mixture. Activity data were plotted as percent residual activity, where 100% activity was taken from enzyme samples incubated without a compound.

#### 3.2.2. Enzymatic Inhibition Assays of the G6PD::6PGL Protein with Selected Compounds

The IC_50_ values of the four selected compounds were determined. The IC_50_ has been established as the compound concentration at which the enzyme loses 50% of its activity. The assay was performed using a protein concentration of 0.2 mg/mL in buffer T (100 mM Tris–HCl, 30 mM MgCl_2_, pH 8) with different concentrations of the compounds (0 to 800 µM). The protein was incubated at 37 °C for 2 h after the residual activity of the G6PD domain was measured. The data were plotted as percent activity, where 100% was taken as the enzyme’s activity without compounds. All values were adjusted to the Boltzmann sigmoid equation in the Origin 8.0^®^ program (Northampton, MA, USA) to obtain the IC_50_ value expressed in µM units.

#### 3.2.3. Second-Order Inactivation Constant (*k*_2_) of G6PD::6PGL from *Giardia lamblia*

Inactivation assays were performed to determine the rate of each to form the enzyme–inhibitor complex. First, the pseudo-first-order constants (*k*_1_) were obtained by incubating 0.2 mg/mL of pure G6PD::6PGL enzyme and different concentrations of compound (0 to 500 µM) at 37 °C in time intervals (0–120 min). After the incubation period, enzyme activity was measured spectrophotometrically following the reduction of NADP^+^ at 340 nm for each experimental condition. The results were expressed in terms of the activity percentage against each compound’s concentration. The residual activity data were fitted to a mono-exponential decay model, using the equation $A_{R}=A_{0}\;e^{-kt}$, where A_R_ is the residual activity at different times, A_0_ is the activity at the initial time, and *k* is the pseudo-first-order inactivation constant (min^−1^). Subsequently, the constants obtained were plotted against the corresponding inhibitor concentration. A linear regression model was applied to obtain the second-order inactivation constant *k*_2_ (M^−1^ s^−1^) [22,24,46].

### 3.3. Structural Studies of the G6PD::6PGL Fused Enzyme Exposed Inhibitory Compounds

#### 3.3.1. Circular Dichroism (DC) Assays

Secondary structure analysis of the recombinant G6PD::6PGL fused protein incubated with the inhibitors was performed by circular dichroism (CD) in a spectropolarimeter (Jasco J-810^®^, Inc., Easton, MD, USA), equipped with a thermostated Peltier cell support, with a constant passage of high-purity nitrogen. Secondary structure determination was performed in the far-UV region (190–250 nm) at 1 nm intervals in a quartz cuvette with a path length of 0.1 cm. The assays were performed at a protein concentration of 0.4 mg/mL in 50 mM phosphate buffer, pH 7.35, and previously incubated at the IC_50_ of each compound at 37 °C for 2 h. Spectra of the 50 mM phosphate buffer (pH 7.35) containing each compound were used as blanks and subtracted from all the obtained spectra containing the fused parasite enzyme [22,24,46].

#### 3.3.2. Intrinsic and Extrinsic Fluorescence Assays

Intrinsic and extrinsic fluorescence assays have been widely used to monitor changes in the tertiary structures of proteins in the presence of inhibitors [24,46,47]. Fluorescence assays were performed by adjusting the protein to a concentration of 0.1 mg/mL and incubating for 2 h at 37 °C and with the IC_50_ concentration of the inhibitor. Both assays were performed on a Perkin Elmer LS-55 fluorescence spectrometer (Perkin Elmer, Wellesley, MA, USA). For the intrinsic fluorescence assay, the samples were excited at 295 nm, with excitation and emission slits of 4.0 nm and 5.0 nm, respectively, and the emission spectra were obtained from 300 to 500 nm. For the extrinsic fluorescence assay, 8-anilinonaphthalene-1-sulfonic acid (ANS) was used, and the samples were excited at 395 nm using slits of excitation and emission of 4.0 and 5.0 nm, respectively; then, the emission spectra were recorded from 400 to 600 nm.

### 3.4. Molecular Docking Method to Estimate the Binding Energy

The binding mode of compounds CNZ-7, CNZ-8, CMC-1, and FLP-2 with G6PD::6PGL protein of the *Giardia lamblia* was studied using the SwissDock web service (Swiss Institute of Bioinformatics, Lausanne Suisse) [48]. The model of the target protein was generated using the AlphaFold2 notebook [49], implemented in the ColabFold Google project (Seoul, Republic of Korea) [50]. The protein structure was further optimized for the docking study using the protein preparation wizard of the Maestro molecular modeling interface (Schrodinger). The missing hydrogen atoms were added to the structure, and the hydrogen bond network was optimized using MolProbity (Manchester, England) [51]. Structures of CNZ-7, CNZ-8, FLP-2, and CMC-1, drawn using ChemSketch software (Advanced Chemistry Development, Inc. Toronto, ON, Canada), were converted to 3D structures with the help of the Avogadro 1.2.0 tool. Molecular Mechanics Force Field 94 (MMFF94) and 500 steps were used for the geometrical optimization of ligands to obtain the most stable structure with the lowest possible ground state energy; therefore, it was used for a detailed description of geometrical optimization. The results were visualized and analyzed in Chimera UCSF (San Francisco, CA, USA) [52] to select the pockets with the lowest binding energy (kcal/mol), the highest percentage of poses, and H-bond interactions.

### 3.5. In Vitro Assays

#### 3.5.1. Antigiardial Activity

The *Giardia lamblia* WB strain (ATCC number 30957) was grown and cultivated in a modified TYI-S-33 medium supplemented with 10% fetal bovine serum at 37 °C. *G. lamblia* was harvested from a confluent culture by chilling the tubes on ice for 5–10 min to detach cells, followed by centrifugation at 800× *g* for 5 min. For the antigiardial activity assay, 5 × 10^4^ trophozoites of *G. lamblia* were incubated with different concentrations of the compounds (31, 62, 125, 250, and 500 µM) in a final volume of 1.5 mL and then incubated for 48 h at 37 °C. After incubation, 75 µL of the treated trophozoites were subcultured individually for another 48 h in a fresh medium without compounds. At the end of this period, the viable trophozoites were counted using trypan blue. Each compound was assayed in three independent experiments. Appropriate controls were tested in each assay, including a sample without compounds and another with only 2% DMSO.

#### 3.5.2. In Vitro Cytotoxicity

The cytotoxicity assay was performed using human cell lines Caco-2 (ATCC HTB-37) and HT29 (ATCC HHTB-38) with a Cell Proliferation Kit II (XTT; St Louis, MO, USA). A cell suspension of 2 × 10^5^ cells/mL was prepared in Dulbecco’s modified Eagle’s (DMEM) medium, supplemented with 10% inactivated fetal bovine serum and 1% penicillin/streptomycin (5000 U/mL), and 100 µL portions of the suspension were added to the wells of 96-well plates. The plates were incubated for 24 h at 37 °C in an atmosphere of 5% CO_2_. The medium in each well was replaced with 100 µL medium with compounds at 500, 250, 150, 75, 32, and 16 µM. After 48 h of incubation at 37 °C in an atmosphere of 5% CO_2_, the viability of cells was determined. The medium in each well was gently replaced with 100 µL of fresh medium, 50 µL of XTT labeling mixture was added per well, the mixture was incubated for four h at 37 °C and 5% CO_2_, and the absorbance at 495 nm was measured. Each compound was assayed in triplicate via three independent experiments. The CCI_50_ values were determined using the nonlinear regression function of GraphPad Prism version 6.04 for Windows.

## 4. Conclusions

In this work, we identified four compounds that inhibit the catalytic activity of the fused protein G6PD::6PGL from *Giardia lamblia*. Second-order constants showed that CMC-1 and CNZ-7 presented the highest reactivity, 0.97 and 1.8 M^−1^ s^−1^, respectively. Moreover, structural studies indicated that the loss of the catalytic activity of the fused G6PD::6PGL protein was caused by the interaction of the compounds, which affects both the secondary and tertiary structures. In addition, we identified the potential interaction sites by molecular docking. We found that the compounds (CNZ-7, CNZ-8, CMC-1, and FLP-2) bind near the binding sites for the G6P and NADP^+^ substrates of the G6PD domain and the structural NADP^+^ binding site. The next step was to test these compounds directly on *G. lamblia* trophozoites to verify their inhibitory effect and the impact on their viability. After calculating the IC_50_ for each compound, it was evident that CNZ-7, CNZ-8, and FLP-2 required low concentrations (IC_50_: 8.7, 15.2, and 15.3 µM, respectively) to decrease the trophozoites’ viability. Finally, these compounds require high concentrations (500 µM) to induce a cytotoxic effect on the Caco2 and HT-29 cell lines, which is a good indicator of the selectivity of the compounds. With the data shown in the present study, the compounds CMC-1, CNZ-7, CNC-8, and FLP-2 are proposed as inhibitors of the G6PD::6PGL enzyme of *G. lamblia* and are an attractive potential starting point for new experimental antigiardial drugs.

## Figures and Tables

**Table 1:** The compounds from an in-house-library show inhibition of more than 70% on the fused G6PD::6PGL enzyme at a final concentration of 400 μM.

Compounds	G6PD::6PGL Inhibition (%) at (400 μM)	HsG6PD Inhibition (%) at (400 μM)	G6PD::6PGL IC_50_ (μM)
CNZ-7	85	68	150
CNZ-8	71	43	80
CMC-1	98	34	70
FLP-2	90	20	256
Nitazoxanide	100	ND	78
Barbituric acid	0	ND	>500

ND. Not determined.

**Table 2:** The interaction energy between the studied ligands and the G6PD::6PGL protein. H-Bond is the hydrogen bond interaction; ΔG is the free energy of binding in kcal/mol.

Compounds	Substrates Binding Site		Structural NADP^+^ Binding Site	
	H-Bond	ΔG (kcal/mol)	H-Bond	ΔG (kcal/mol)
CNZ-7	4	−7.47	3	−8.62
CNZ-8	3	−7.06	6	−7.60
CMC-1	4	−6.55	2	−7.22
FLP-2	2	−7.11	1	−7.66

**Table 3:** In vitro giardicidal activity and selective cytotoxicity shown by selected compounds.

Compounds	IC_50_ (µM)	CC_50_ (µM)	
	*G. lamblia*	HT29 (SI)	Caco-2 (SI)
CNZ-7	8.7	529 (60)	640 (74)
CNZ-8	15.2	622 (41)	633 (41)
FLP-2	15.3	1912 (125)	3184 (208)
CMC-1	24.1	3218 (134)	4006 (166)
Metronidazole	4.8	265.9 (52) [38]	19 (3.9) [39]
Nitazoxanide	4.2	>50 (12) [40]	26.8 (7) [41]

The numbers inside the parentheses correspond to the ratio of CC_50_/IC_50_ values between mammalian cells and trophozoites (selectivity index).

**Table 4:** Pharmacokinetic predictive values calculated with ADMETLab 2.0 for compounds CNZ-7, CNZ-8, FLP-2, CMC-1, nitazoxanide, and barbituric acid.

Model		Compounds					
		CNZ-7	CNZ-8	FLP-2	CMC-1	Nitazoxanide	Barbituric Acid
A	Gastrointestinal absorption	(+++)	(+++)	(+++)	(+++)	(+++)	(---)
	Blood–brain barrier permeation	(+)	(+)	(+++)	(+++)	(+)	(--)
D	Plasma protein binding	93%	81.4%	62.8%	96.7%	67.0%	32.9%
	Volume distribution	0.68 L/kg	0.824 L/kg	0.59 L/kg	0.337 L/kg	0.541 L/kg	0.521 L/kg
M	CYP3A4 substrate	(-)	(--)	(--)	(--)	(--)	(---)
	CYP2D6 substrate	(++)	(-)	(--)	(-)	(--)	(---)
E	Clearance	5.69 mL/min/Kg	2.91 mL/min/Kg	6.6 mL/min/Kg	0.51 mL/min/kg	2.14 mL/min/kg	2.14 mL/min/kg
	Half-life (T_½_)	>3 h	>3 h	>3 h	>3 h	>3 h	>3 h
T	hERG blockers	(--)	(---)	(---)	(--)	(---)	(---)
	Rat oral acute toxicity	(--)	(--)	(++)	(---)	(+++)	(---)
	Carcinogenicity	(+++)	(+++)	(+++)	(--)	(+++)	(--)

The prediction probability values are transformed into six symbols: 0–0.1 (---), 0.1–0.3 (--), 0.3–0.5 (-), 0.5–0.7 (+), 0.7–0.9 (++), and 0.9–1.0 (+++).

## Data Availability

Not applicable.

## Supplementary Materials

The following supporting information can be downloaded at: https://www.mdpi.com/article/10.3390/ijms232214358/s1, Figure S1: Purification of the recombinant GlG6PD::6PGL enzyme.
