# Supplementary material for: Giardia lamblia G6PD::6PGL Fused Protein Inhibitors Decrease Trophozoite Viability: A New Alternative against Giardiasis
Source: Int J Mol Sci. 2022 Nov 18;23(22):14358. doi: 10.3390/ijms232214358 (PMC9697976; doi:10.3390/ijms232214358)
Supplement: Supplementary file 1 [file ijms-23-14358-s001.zip › ijms-1967599-supplementary.pdf]

Article

Giardia lamblia G6PD::6PGL Fused Protein Inhibitors Decrease Trophozoite Viability: A New Alternative against Giardiasis

Laura Morales-Luna [1,2], Beatriz Hernández-Ochoa [3], Víctor Martínez-Rosas [1,4], Gabriel Navarrete-Vázquez [5], Daniel Ortega-Cuellar [6], Yadira Rufino-González [7], Abigail González-Valdez [8], Roberto Arreguin-Espinosa [9], Adrián Marcelo Franco-Vásquez [9], Verónica Pérez de la Cruz [10], Sergio Enríquez-Flores [11], Carlos Martínez-Conde [5], Luis Miguel Canseco-Ávila [12], Fernando Gómez-Chávez [13] and Saúl Gómez-Manzo [1,*]

[1] Laboratorio de Bioquímica Genética, Instituto Nacional de Pediatría, Secretaría de Salud, Mexico City 04530, Mexico
[2] Posgrado en Ciencias Biológicas, Universidad Nacional Autónoma de México, Mexico City 04510, Mexico
[3] Laboratorio de Inmunoquímica, Hospital Infantil de México Federico Gómez, Secretaría de Salud, Mexico City 06720, Mexico
[4] Programa de Posgrado en Biomedicina y Biotecnología Molecular, Escuela Nacional de Ciencias Biológicas, Instituto Politécnico Nacional, Mexico City 11340, Mexico
[5] Facultad de Farmacia, Universidad Autónoma del Estado de Morelos, Av. Universidad 1001, Chamilpa, Cuernavaca, Morelos 62209, Mexico
[6] Laboratorio de Nutrición Experimental, Instituto Nacional de Pediatría, Secretaría de Salud, Mexico City 04530, Mexico
[7] Laboratorio de Parasitología Experimental, Instituto Nacional de Pediatría, Secretaría de Salud, 04530 Ciudad de México, México
[8] Departamento de Biología Molecular y Biotecnología, Instituto de Investigaciones Biomédicas, Universidad Nacional Autónoma de México, Mexico City 04510, Mexico
[9] Departamento de Química de Biomacromoléculas, Instituto de Química, Universidad Nacional Autónoma de México, Mexico City 04510, Mexico
[10] Neurobiochemistry and Behavior Laboratory, National Institute of Neurology and Neurosurgery "Manuel Velasco Suárez", Mexico City 14269, Mexico
[11] Laboratorio de Biomoléculas y Salud Infantil, Instituto Nacional de Pediatría, Secretaría de Salud, Ciudad de México 04530, Mexico
[12] Facultad de Ciencias Químicas, Campus IV, Universidad Autónoma de Chiapas, Tapachula City 30580, Mexico
[13] Laboratorio de Enfermedades Osteoarticulares e Inmunológicas, Sección de Estudios de Posgrado e Investigación, Escuela Nacional de Medicina y Homeopatía, Instituto Politécnico Nacional, Mexico City 07320, Mexico
* Correspondence: saulmanzo@ciencias.unam.mx; Tel.: +52-55-1084-0900 (ext. 1442)

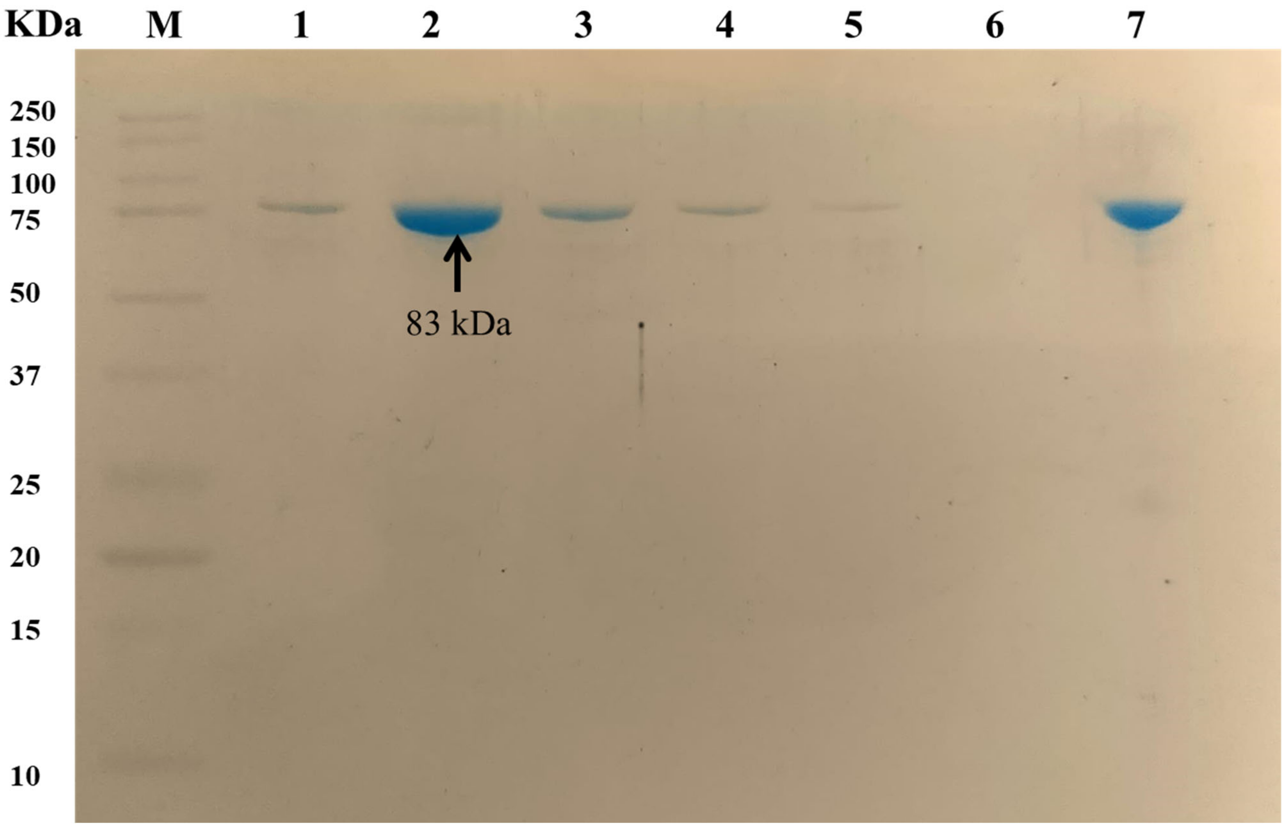

Figure S1. Purification of the recombinant GlG6PD::6PGL enzyme. M: molecular protein weight (MW) marker precision plus protein kaleidoscope standards from Bio-Rad. Lines 1 - 6: 15 µL of protein fractions showed G6PD activity. Line 7 shown the purified protein. The gel was stained with a colloidal Coomassie solution. The SDS-PAGE gels are representative of three independent experiments.
